# Supplementary material for: Complexome profiling of the Chlamydomonas psb28 mutant reveals TEF5 as an early PSII assembly factor
Source: Plant Cell. 2025 Mar 17;37(6):koaf055. doi: 10.1093/plcell/koaf055 (PMC12164590; doi:10.1093/plcell/koaf055)
Supplement: koaf055_Supplementary_Data [file koaf055_supplementary_data.zip › psb28_interactive.zip!/plots/intensity/qualityPlots/peptides/POR1_Cre01.g015350.t1.1.html]

### Cre01.g015350.t1.1

Trivial name: POR1  
SeqCoverage [median]: 24.70  
Mass: 41.87 kDa  
  
 

| Sequence | MissedCleavage | Mass [Da] | wtIntensity | muIntensity | Score |
| --- | --- | --- | --- | --- | --- |
| EHVPLFK | 0 | 868.48 | 10997.63 | 0.00 | 106.76 |
| GYVSEEEAGR | 0 | 1095.48 | 5662.58 | 0.00 | 101.43 |
| GYVSEEEAGRR | 1 | 1251.58 | 75167.72 | 37908.13 | 150.10 |
| LAAVISDPK | 0 | 912.53 | 127497.84 | 49916.52 | 129.47 |
| LAAVISDPKLNK | 1 | 1267.75 | 41183.51 | 7443.56 | 138.71 |
| LVGLSA | 0 | 558.34 | 4492.62 | 2775.69 | 93.15 |
| LWDISAK | 0 | 831.45 | 1340.14 | 0.00 | 81.30 |
| QFVQNFK | 0 | 909.47 | 11182.49 | 0.00 | 129.96 |
| QTAIITGASSGLGLNAAK | 0 | 1671.92 | 50074.31 | 16914.05 | 228.17 |
| TLFPPFQK | 0 | 976.54 | 10151.24 | 0.00 | 75.31 |
| VGMPAGSYSILHLDLSSLESVR | 0 | 2330.19 | 1476.97 | 0.00 | 48.62 |
|  |  |  | 339227.0 | 114958.0 |  |
